# Testing whether paralinguistic cues alter math learning in highly math anxious adults

**DOI:** 10.3389/fpsyg.2026.1752172

**Published:** 2026-06-17

**Authors:** Bijan I. Tabrizian, Ian M. Lyons

**Affiliations:** Department of Psychology, Georgetown University, Washington, DC, United States

**Keywords:** adult math education, math anxiety, math avoidance, math performance, paralinguistic cues

## Abstract

**Introduction:**

Recent discovery of a “math anxiety transference” effect – wherein math anxious instructors produce underperforming learners, who are then more prone to math anxiety themselves – threatens to undercut math education’s provision of individually- and societally-vital numeracy skills. While previous theorists have explained this transference using *person-* and *content-based* explanations, the present work explores a *communication-based* perspective via the novel Paralinguistic Model of Math Anxiety Transference. This model posits that math anxiety influences the way teachers *speak* during math instruction, producing anxious paralinguistic cues that, when detected by learners, produce appraisals of instructor anxiety, then elevated learner state anxiety, and finally worsened math performance and math avoidance.

**Methods:**

Data from *N* = 86 highly-math-anxious, adult participants were collected via Prolific across two sessions, with session two exposing participants to either paralinguistically anxious (*n* = 46) or paralinguistically confident (*n* = 40) math instruction.

**Results:**

Results indicated that paralinguistically-anxious teachers [H_1_] were rated as more anxious, but [H_2_] did *not* produce elevated learner state anxiety, and [H_3a-b_] did not produce differential changes in math performance or avoidance, relative to their paralinguistically-confident counterparts. Rather, participants in *both* the anxious-instructor and confident-instructor groups exhibited significant improvements in math performance and significant reductions in math avoidance from S1 to S2. We also tested for the role of time–pressure, which did not interact with the above effects.

**Discussion:**

These results suggest that math anxious adult educators needn’t be worried that their potentially-anxious speech will interfere with students’ ability to learn. Moreover, they propose a low-cost, easily distributable medium of effective adult math education in an age of declining numeracy.

## Introduction

Across traditional and adult-educational contexts, high quality math instruction serves as a foundation of joint personal and societal development. For individual learners, it plays a pivotal role in facilitating human-, social-, and identity-capital development [i.e., boosted employability (Durrani and Tariq, 2012), bolstered social networks (Balatti et al., 2006), improved self-confidence (Swain, 2005), etc.]. At the societal level, it contributes to a competitive STEM workforce (National Center for Science and Engineering Statistics, 2024), predicts economic growth (Hanushek et al., 2008; Geimer, 2014), and thus maintains global competitiveness amidst an age of rapid technological and empirical advancement. It is by virtue of this importance that any threat to math education’s effectiveness should be responded to with prompt investigation. One such threat – uncovered relatively recently and supported by a robust body of research – lies in the tendency for math anxious instructors (i.e., those who are anxious about math and/or teaching math) to produce learners with significantly lower levels of math performance. This math anxiety (MA) transference effect has been replicated across learner ages, learner and instructor genders, geographical regions, and income statuses (Beilock et al., 2010; Hadley and Dorward, 2011; Ramirez et al., 2018; Richland et al., 2020; Schaeffer et al., 2021; Szczygieł, 2020), suggesting that the scope of the problem is rather large. Moreover, while the capacity of these math anxious instructors to compromise their learners’ math achievement already constitutes a matter of relative urgency, math performance’s bidirectional, longitudinal relationship with math anxiety amplifies said urgency even further. Namely, the cyclicality of this relationship – with deficits in math performance producing long-term increases in math anxiety (Kyttälä and Björn, 2010; Ma and Xu, 2004; Sorvo et al., 2019; Szczygieł et al., 2024; Zhang et al., 2023), which then produce further reductions in math performance (Carey et al., 2016; Cargnelutti et al., 2017; Sorvo et al., 2022) – extends the performance-based harm imposed by math anxious instructors into the future. Put simply, by harming learners’ performance, math anxious instructors become the first dominoes down a self-reinforcing path toward debilitating math anxiety and an inability to complete even the simplest of math tasks. With the joint criticality and universality of this threat established, its present investigation is warranted.

Existing literature on the MA transference effect has offered a handful of characterizations for its central instructor-MA-to-learner-performance pathway. The majority of these proposals center around two themes. On one hand, there are those which highlight MA’s influence over the academic resources that instructors provide to learners. These *content-based* mechanisms center factors like reduced math instructional duration (Schaeffer et al., 2021), harsher math-task feedback (Ramirez et al., 2018), and an adherence toward ineffective teaching practices (Hadley and Dorward, 2011; Ramirez et al., 2018) within math anxious instructors’ classrooms, explaining performance reductions via a deprivation of the resources needed to thrive. Alongside these explanations are their *person-based* counterparts, which pin faltering math performance not on the resources provided by instructors, but on the *instructors themselves*. These *person-based* theories propose that something about math anxious instructors [e.g., their promotion of beliefs that math is unimportant and/or intimidating (Schaeffer et al., 2021; Szczygieł, 2020)], their fixed mindsets regarding math ability (i.e., that it is innate rather than effort-based) (Ramirez et al., 2018; Szczygieł, 2020) incites reduced learner math engagement, ultimately predicting performance deficits.

**Figure 1:**
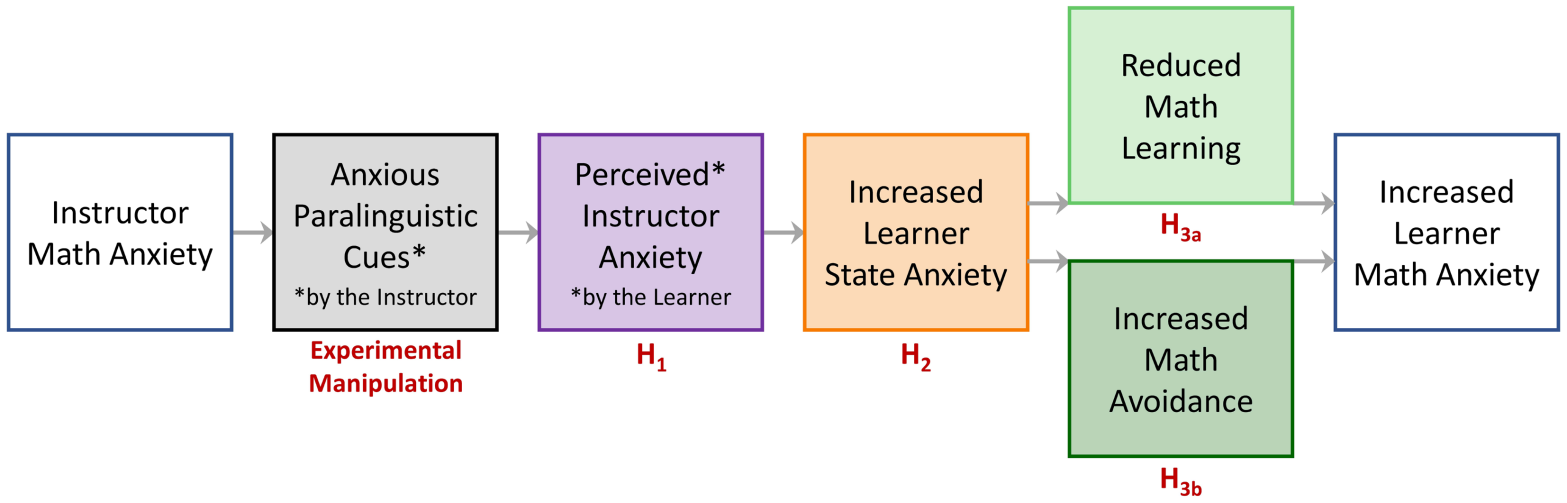
Visualizes the paralinguistic model (PM) of how the math anxiety of an instructor can impact a learner. Lettering in red indicates aspects of the model directly manipulated or measured in this study. Color-coding of specific boxes serve as reference points for the remainder of the paper.

While these explanations, taken together, cover a broad swath of the classroom environment, there remains one piece of the puzzle which has until now remained unexplored. Namely, the present work argues for instructor math anxiety’s *communication-based* impact, centering the *paralinguistic* aspects of math instruction in explaining instructors’ performance-based influences. Put simply, rather than attributing performance deficits to *what* is taught (content-based) or *who* is teaching it (person-based), this approach focuses on *how* math concepts are being communicated to learners. In grounding this approach, the following section explores a novel theoretical pathway, connecting instructor math anxiety to learner math performance via paralinguistic cue usage, affective appraisal, and state anxiety changes. In the current study, we propose a Paralinguistic Model (PM) to describe how an instructor or teacher’s emotional state can potentially be transferred to that of a learner, possibly also impacting learning outcomes as well. This model is visually summarized in Figure 1, and the next section provides a detailed explanation and empirical support for each of the hypothesized steps therein.

### The paralinguistic model of math anxiety transference

The term *paralinguistic* refers to the components of spoken communication which carry meaning without possessing any lexical definition. Common paralinguistic cues include changes in intonation, pitch, pause usage, and speaking speed, each of which work together in communicating meaning beyond the words being spoken. These cues gain relevance within the present model, hereafter referred to as the Paralinguistic Model (PM), when one considers their ability to reflect speakers’ internal affective states. Namely, an extensive body of research supports that individuals, when speaking in contexts that make them anxious, reliably exhibit a distinct set of paralinguistic cues (i.e., higher pitch, increased pause frequency, vocal shakiness, increased vocal stumble incidence) as a result of this anxiety (Brück et al., 2011; Laukka et al., 2008; Lewin et al., 1996; Mulac and Sherman, 1974; Paeschke, 2004; Weeks et al., 2012). Thus, when math-anxious instructors speak about math in the classroom, there is empirical support for their likely use of this “anxious voice” (gray box in Figure 1).

Having supported math-anxious instructors’ use of an anxious voice, this voice is then *recognized* as anxious by the learners listening to it. The literature on affective vocal recognition provides this support for this phenomenon, demonstrating that audiences listening to the same paralinguistic cues associated above with anxious situations tend to rate the accompanying speaker higher on gauges of anxiety and nervousness (Carli et al., 1995; Laukka et al., 2008). Within the classroom context, this evidence suggests that math anxious instructors’ use of “anxious voice” will contribute to their learners’ appraisal of the instructor as anxious (purple box in Figure 1).

From here, evidence suggests a relationship between this anxious vocal recognition and increased learner state anxiety. While previous work has uncovered positive associations between instructor and learner state anxiety (Sinclair and Ryan, 1987), this work fails to support vocal appraisal’s role in mediating the relationship. However, this role is supported by more recent contributions to the affective appraisal literature. On one front, researchers have found a positive association between the state anxiety levels of speakers and listeners within a classroom context (Behnke et al., 1994), supporting the relevance of speech-based factors within the initial association. On another front, a recent investigation found that learner perceptions of instructor anxiety are “emotionally contagious,” resulting in elevated levels of state anxiety for the learners themselves (Amiri, 2024). Taken together, these sources support (A) that instructor and learner state anxieties are positively associated, (B) that spoken communication is a relevant mechanism for this association, and (C) that learner perceptions of anxiety likely mediate this association. Thus, after “anxious voice” usage contributes to learner perceptions of an instructor as anxious, a case can be made for the role of this voice-based perception in contributing to a learner state anxiety increase (orange box in Figure 1).

With this increase in learners’ state anxiety, it becomes important to observe potential behavioral outcomes, such as reduced math learning and increased math avoidance (light and dark green boxes in Figure 1, respectively). Turning first to math learning, one line of evidence suggesting a connection to state anxiety stems from interventional literature on math performance. Within this work, factors responsible for inciting significant increases in math performance (i.e., humor-based interventions, dispositional mindfulness) were found to achieve these increases by first acting on (i.e., alleviating) state anxiety (Bellinger et al., 2015; Ford et al., 2012; Sammallahti et al., 2023). This mediated line of evidence is further supported by investigations uncovering a direct negative association between state anxiety and math performance (Meijer, 2001; Orbach et al., 2020) – one which is likely driven by state anxiety’s limitation of cognitive resources via its heightening of threat-based attentional biases (Meijer, 2001). Thus, when learner perceptions of instructor anxiety contribute to elevated state anxiety levels, evidence suggests that math performance will suffer as a result. What is less clear, however, is whether this increased anxiety impacts math *learning* (light green box in Figure 1). There is evidence that math anxiety can impact math learning with automated feedback, but it is less clear if learning is impacted among math anxious individuals when they encounter an instructor whom they perceive as math anxious.

The present analysis also sought to examine the behavioral impact of increased learner state anxiety on math *avoidance* (Figure 1, dark green box). As was the case with math performance, prior findings suggest that math avoidance is predicted by changes in state anxiety, with increases in state anxiety being associated with increased math avoidance (Orbach et al., 2019). While the present work sought to confirm this relationship, we were doubly interested in examining math avoidance, particularly in light of its mediational effects. Namely, math avoidance is widely reported as a significant (by some accounts, the most significant) mediator in math anxiety’s negative relationship with math performance (Daker et al., 2021; Lyons et al., 2025). This manifests in high-math-anxious individuals’ tendency to engage in “short-term avoidance,” wherein they avoid more effortful (i.e., more rewarding) study strategies in favor of their easier, less rewarding counterparts. This ultimately produces poorer performance outcomes (Choe et al., 2019; Jenifer et al., 2023; Jenifer et al., 2022). Given the highly-math-anxious nature of the present study’s sample, examining math avoidance outcomes may help to contextualize any emergent performance effects. Thus, although the present analysis does not directly interrogate the longitudinal implications of math anxiety transference, its observation of paralinguistic factors’ influence over math avoidance will provide valuable insights for future researchers within the field.

### Current study

In the current study, we manipulated the paralinguistic affect of a math instructor during math instruction to convey either anxiety or confidence on the part of the instructor. Because the paralinguistic model is primarily about math anxious learners, we focused on learners who self-identified as math anxious. This approach allowed us to measure the potential negative impact of a math instructor’s anxious paralinguistic cues on learning-related outcomes in math anxious learners. In so doing, we sought to test several relationships comprising the paralinguistic model. Specifically, we tested the following primary hypotheses. Paralinguistically anxious instructional cues will lead to [H_1_] higher learner ratings of perceived instructor anxiety (Figure 1, purple); [H_2_] higher state anxiety in learners (Figure 1, orange box); [H_3a_] reduced math learning (Figure 1, light green box); [H_3b_] increased math avoidance (Figure 1, dark green box).

In addition, *post hoc* analyses tested whether results for math learning (H_3a_) and math avoidance (H_3b_) varied as a function of time–pressure. Some have argued that highly math anxious individuals are especially susceptible to time–pressure on math tasks, and that time–pressure may undercut learning as well as performance (Boaler, 2012; Boaler, 2014). Hence, we present math items in both timed and untimed settings (both before and after instruction). We can then examine whether the impact of an instructor’s anxious paralinguistic cues on math learning and math avoidance differs as a function of whether the instructed math is performed with or without time pressure. Note that primary analyses average across time pressure conditions to maximize statistical power and external validity; post hoc analyses examine timed and untimed conditions separately.

## Materials and methods

### Procedure

Participants completed two sessions (S1, S2). Both sessions were collected online via the Prolific data collection platform (Douglas et al., 2023). Sessions were separated by an average of 66.14 days (*s* = 6.71). Participants completed a set of modestly challenging math problems in both sessions. Participants completed the same types of math problems in both sessions, though specific problems were not repeated (see *Tasks and Stimuli* below for additional details on specific math problem types and stimuli).

In S1 only, participants also completed a short battery of psychological assessment questionnaires, including a standard math anxiety assessment, and a basics demographics questionnaire – in that order.

S1 and S2 lasted an average of 31.07 and 69.17 min, respectively. Participants were paid an average of $10.01 and $11.63 for completing S1 and S2, respectively. All stimuli and procedures were approved by the Georgetown University Institutional Review Board (IRB).

### Manipulation of paralinguistic cues

Prior to completing math problems in S1, we provided only general paradigm information (e.g., how to enter a response, advance the program, general definitions, etc.). Prior to completing math problems in S2, participants were shown an instructional audio recording that provided step-by-step instructions and worked examples of how to complete each type of math problem (of those assessed here). We manipulated paralinguistic affect by presenting two versions of the instructional video. Participants were randomly assigned to listen to one of two recordings – one in which the instructor spoke with a highly anxious paralinguistic affect, and the other in which they spoke with a highly confident paralinguistic affect (see *Tasks and Stimuli* below for additional video details).

### Key measures and operationalization of hypotheses

After watching the video, participants rated the speaker’s anxiety. We tested H_1_ (Figure 1, purple) by examining differences in participant-rated speaker-anxiety between paralinguistic conditions (S2 only).

After completing the math problems in each session, participants rated their own anxiety state. We tested H_2_ (Figure 1, orange) by examining whether changes in self-rated participant-anxiety ratings from S1 to S2 depended on paralinguistic condition.

We tested H_3a_ (Figure 1, light green) by examining whether changes from S1 to S2 in the number of problems each participant completed correctly depended on paralinguistic condition.

Participants were allowed to skip problems, which we operationalized as a form of behavioral avoidance (Choe et al., 2019; Jenifer et al., 2023). We tested H_3b_ (Figure 1, dark green) by measuring whether changes in the number of skipped problems from S1 to S2 depended on paralinguistic condition.

### Participants

We tested the four primary hypotheses with a final analytic sample of *N* = 86 math anxious individuals (50 female, age: *M* = 44.23, *s* = 14.69).

#### Eligibility criteria and participant selection

Session 1 participation was restricted to registered Prolific users geolocated in the United States. Demographics of the S1 sample were tailored (via Prolific quota procedures) to be aligned with 2024 US census data. Three hundred thirty participants completed all portions of S1. Of those, 45 were removed due to one or more *a priori* exclusion criteria: impaired (and uncorrected) vision or hearing, non-fluent English comprehension, self-identified as having a learning disability.

We next determined which of these 285 participants was eligible for S2. The paralinguistic model primarily concerns *math anxious learners*, so we restricted our S2 sample to those with math anxiety scores in the 50th percentile or higher. In addition, because we are focused here on *math learning*, we also restricted our S2 sample to participants whose math performance scores were at or below the 50th percentile in S1 (If participants scored near ceiling in S1, there was little room for them to demonstrate learning from S1 to S2, at least based on the variable operationalization used here). Together, these restrictions yielded 122 participants eligible for S2. All 122 were invited to participate in S2, of whom 86 accepted (70.5% acceptance rate).

S2-eligible participants were pseudo-randomly assigned to either the anxiety or confidence paralinguistic condition (*n* = 61 to each condition). Pseudo-random assignment was achieved by first dividing all participants into a 5×5 matrix based on S1 math anxiety and performance percentiles. Within each of the 25 cells, random number generation then assigned each member to one of the two paralinguistic conditions. This ensured equal representation of different math anxiety and math ability levels in each paralinguistic condition. Random differences in response-rates (participants were not informed of the condition to which they had been assigned) led to minor sample differences in the paralinguistic conditions: *n* = 46 anxious, *n* = 40 confident. Nevertheless and importantly, the difference in response-rates did not upset the balance between groups in terms of a priori math anxiety or math ability [Math Anxiety S1 comparison between conditions: *t*(84) = 0.06, *p* = 0.954; Math Ability S1 comparison between conditions: *t*(84) = 1.00, *p* = 0.323].

### Tasks and stimuli

#### Math anxiety (S1 only)

We measured math anxiety via the Short Math Anxiety Rating Scale (SMARS; Alexander and Martray, 1989). Participants rate how much anxiety they would feel across 25 math-related situations (e.g., “Being given a set of addition problems to solve on paper”; “Getting ready to study for a math test”). Scores range from 0 to 100 (higher score indicates higher math anxiety). Though there is no official normative data on this scale, Ashcraft and Kirk (2001) is cited as a common standard (Ashcraft and Ridley, 2005; Lyons and Beilock, 2012a,b), and they reported a mean score of 36, with a standard deviation of 16. The current S1 sample (*n* = 285) closely reflected this (*M* = 34.44, *s* = 22.39). Reliability for this metric is high (*ɑ* = 0.96).

#### Instructional videos and paralinguistic manipulation (S2 only)

Prior to the assigned video, participants first saw a set of standard (written) instructions, which can be found in Appendix A. Timestamps were used to verify that participants watched the video as instructed.

The instructional videos for the Anxiety and Confidence conditions followed an identical, pre-written script. This script (and hence each video) was separated into four separate sections, each of which described how to understand and solve each of the four problem types. These sections were presented in the same order for all participants. Doing so improved the naturalness of the videos, and ensured the two paralinguistic versions were the same in this regard. For each problem-type, the instructor provided and worked through multiple examples. The full script for the videos can be found in Appendix B.

To achieve the desired paralinguistic differences (Anxious vs. Confident) for the two versions of the instructional video, we had a professional vocalist read the provided script in two affective modes: anxious and confident. To minimize differences between the two versions as much as possible, the same vocalist recorded both versions. This practice is in line with previous work across the paralinguistic literature (Scherer et al., 1973). While the vocalist was given a brief formal introduction to the paralinguistic cues associated with anxiety and confidence, this introduction was not determinative. Rather, the vocalist was asked to ultimately rely on their experience and best professional judgment to achieve the desire affective differences in the two recordings. Both recordings were between 20 and 25 min in duration, with natural paralinguistic speech patterns leading to minor differences in duration (Anxious = 24:54, Confident = 23:34). As a final verification step, pilot data (*n* = 40) demonstrated that naïve listeners indeed rated the vocalist in the ‘Anxious’ recording as more anxious [*t*(38) = −11.95, *p* < 0.001], and they rated the vocalist in the ‘Confident’ recording as more confident [*t*(38) = 9.94, *p* < 0.001].

#### Speaker-anxiety assessment (S2 only)

Immediately following the conclusion of the instructional video, participants rated the instructor in terms of their anxiety and confidence: “In your opinion, how [anxious/confident] was the speaker of the audio recording you just listened to?” Two separate ratings were made (one each for anxious and confident) on a 0–7 Likert scale, with a higher value indicating more anxious/confident. The anxious rating served as our measure of the learner rating of Perceived Instructor Anxiety (H_1_).

#### State anxiety (S1 and S2)

In each session, immediately after completing the full block of math problems (see next section), participants rated their current level of anxiety: “How anxious do you currently feel?” Ratings were made on a 1–7 Likert scale, with a higher value indicating more anxious. Prior work has demonstrated that this is a reliable and highly predictive metric of a person’s subjective anxiety state, especially in a math context (Daker et al., 2023). We operationalized Changes in State Anxiety as the difference in post-math state-anxiety ratings between sessions (S2–S1; H_2_).

#### Math performance and learning (S1 and S2)

We sought a metric of adult math competency that reflected several different types of math problem-solving. We avoided hyper-specific and especially difficult math domains (e.g., advanced calculus, linear algebra, etc.), and instead focused on types of math that might reasonably be known or learned by a typical adult. As a starting point and initial foundation, we used Steiner and Ashcraft’s (2012) Brief Math Assessment (BMA). From the BMA, we took four math problem types: (1) multi-step whole-number arithmetic, (2) fraction arithmetic, (3) solving systems of linear equations, and (4) reduction of algebraic expressions. We then increased the number of items in each problem-type to provide more robust measurement of each type, and to allow for problem-types to be repeated across sessions, without having to repeat individual problems. Examples of each problem-type are given in Appendix C; a full list of all problems will be made available on the project’s Open Science Framework page upon publication. General instructions given to participants for this task (in both S1 and S2) are given in Appendix D.

In each session, participants saw six problems of each type (24 items total). To increase external validity, half of the problems were presented under time–pressure (15 min to complete 12 problems, 3 of each problem-type), and half were presented with no time-limit. The order of the different problem-types and presence/absence of time–pressure was counterbalanced. Problems were presented all at once (i.e., on a single screen) and required open-ended responses. Math Performance was operationalized as the net number correct across all 24 problems in a given session. Math Learning was operationalized as the difference in Math Performance between sessions (S2–S1; H_3a_).

#### Math avoidance (S1 and S2)

We operationalized math avoidance as *computational* math avoidance (sometimes referred to as ‘micro-avoidance’; Daker et al., 2021), in which one behaviorally chooses not to engage with or expend effort on what one perceives to be a challenging math task (Choe et al., 2019; Jenifer et al., 2023). Participants were not required to provide an answer to every problem in that they were allowed to skip individual problems. We thus operationalized (computational) Math Avoidance as the number of problems skipped in a given session. We operationalized Changes in Math Avoidance as the difference in skipped problems between sessions (S2–S1; H_3b_).

## Results

### Preliminary analysis: paradigm validation and testing H_1_

#### H_1_: learner perceptions of the instructor

**Figure 2:**
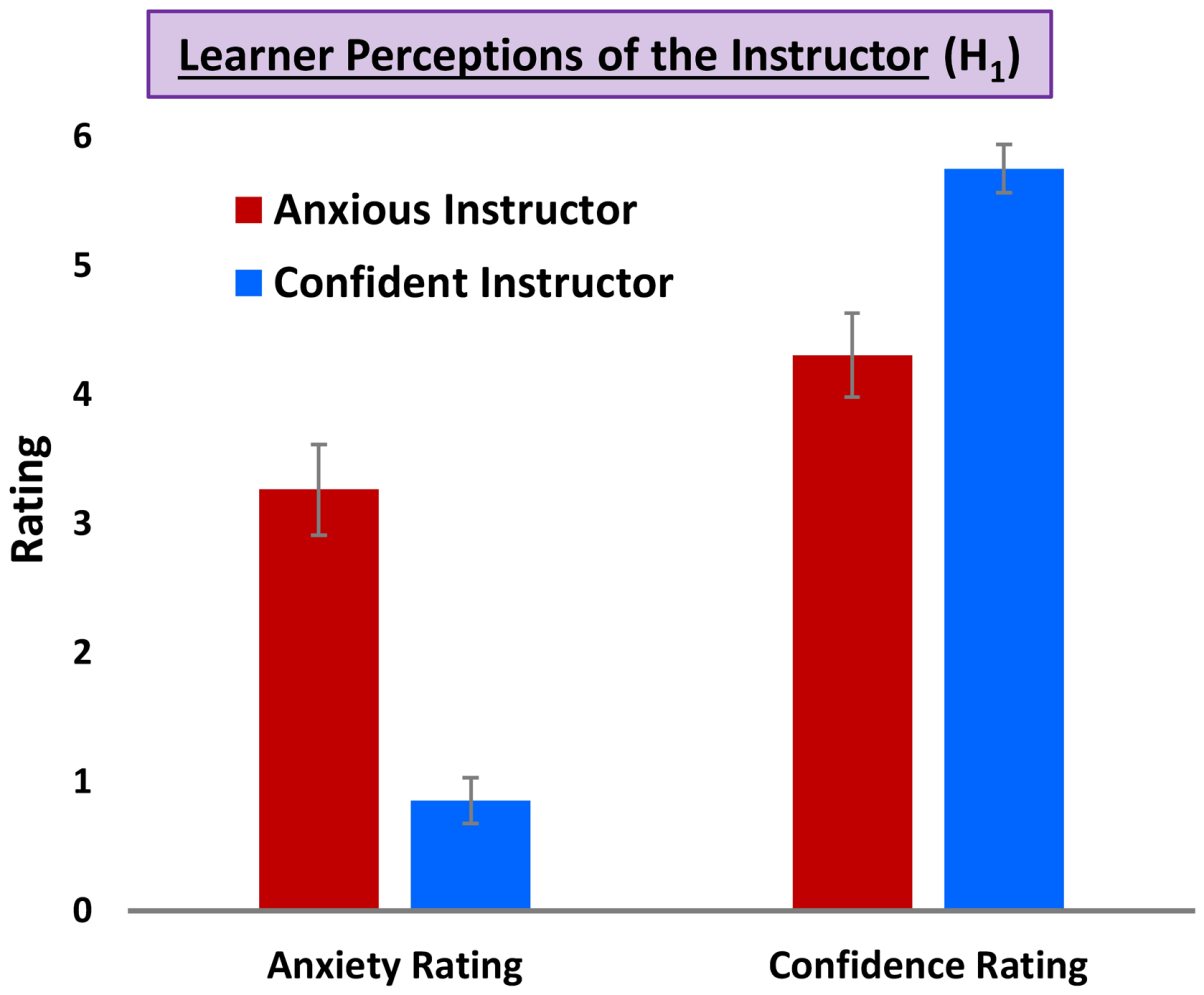
Shows learners’ ratings of the instructor they viewed. Learners rated the instructor who used anxious paralinguistic cues as more anxious, and learners rated the instructor who used confident paralinguistic cues as more confident. Note that comparing the left two bars constitutes a direct test of our first hypothesis (H_1_). Error-bars are standard-errors of the mean.

Here, we tested whether learners rated the instructor in the different paralinguistic conditions per the intended experimental manipulation. Specifically, we expected that learners who heard the paralinguistically ‘anxious’ version of the instructor would rate the instructor as more anxious than learners who heard the paralinguistically ‘confident’ version of the instructor. We also expected the reverse to be true: learners who heard the paralinguistically ‘confident’ version of the instructor would rate the instructor as more confident that learners who heard the paralinguistically ‘anxious’ version. This analysis in effect serves as a manipulation check: Did the different paralinguistic conditions elicit the intended perceptions of the instructor in the learners in each of the respective conditions? At the same time, this analysis tests H_1_ (Figure 1, purple box). Results are visualized in Figure 2.

Learners who heard the instructor with paralinguistically anxious cues rated the instructor as significantly more anxious than learners who heard the instructor with paralinguistically confident cues: *t*(65.70) = 6.09, *p* = <0.001, *d* = 1.26 (We adjusted degrees of freedom because equal variances could not be assumed for this test). Conversely, learners who heard the instructor with paralinguistically confident cues rated the instructor as significantly more confident than learners who heard the instructor with paralinguistically anxious cues: *t*(71.37) = 3.87, *p* < 0.001, *d* = 0.80. The paralinguistic cues impacted learner perceptions of the instructor as intended. We next turn to whether these cues impacted changes in learner anxiety and learning outcomes.

### Primary analyses: impact of paralinguistic cues on changes in learner anxiety (H_2_) and learning outcomes (H_3a_ and H_3b_)

**Figure 3:**
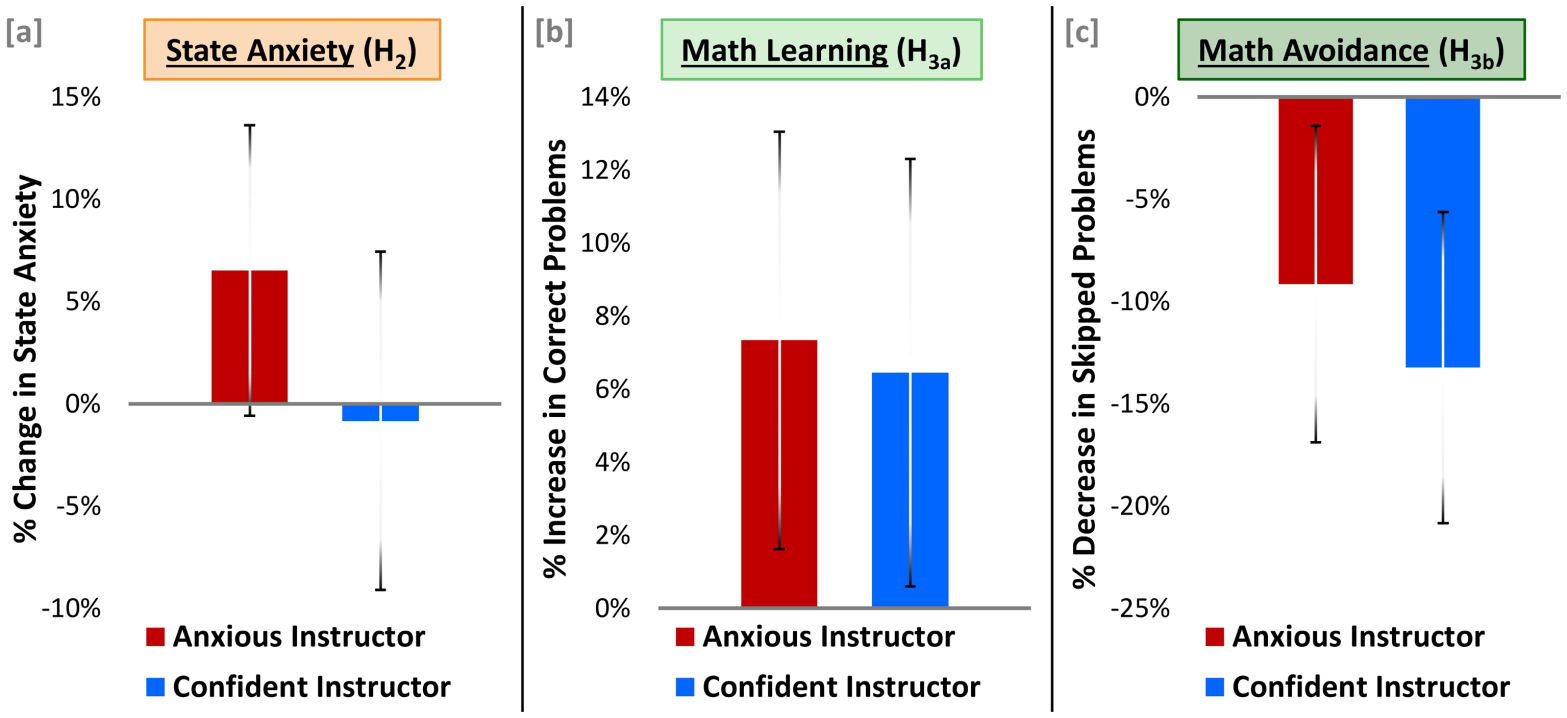
Shows the results of our tests of whether instructor paralinguistic cues modulated learning-related changes in **(a)** state anxiety (H_2_), **(b)** math learning (H_3a_) and **(c)** math avoidance (H_3b_). Primary analyses concern directly comparing the red and blue bar for a given outcome. No comparisons were statistically significant (see text for details). Error-bars are 95% confidence intervals for that condition mean. Thus, if a given interval does not cross 0, this means that there was a significant change between sessions (S2–S1) for that group. For instance, learners who viewed the anxious instructor showed a significant increase in the percentage of problems they answered correctly (red bar, **b**). The negative values in **(c)** mean that there was a reduction in the number of problems that were skipped in S2 relative to S1.

Recall that outcome variables for H_2_, H_3a_ and H_3b_ were operationalized as changes from S1 to S2 (S2–S1). We are primarily interested in whether these changes differed as a function of instructor paralinguistic cues: Anxious vs. Confident condition. The analyses below are thus a between-subjects test comparing changes in the relevant outcome (difference scores between sessions). Note that this is mathematically equivalent to the interaction term of a 2(Session) × 2(Instructor Condition) ANOVA. In other words, we are testing whether the change in a given variable (difference between sessions) *depends on* paralinguistic cues (instructor condition). Results are visualized in Figure 3; for ease of interpretation, change values are converted from native units into percent change. For instance, Figure 3b shows the increase in the percentage of items participants answered correctly (out of 24). For full transparency, we report all cell means in native units in Table 1.

**Table 1:** Mean state anxiety and math performance/avoidance by paralinguistic condition.

State anxiety rating (H_2_)		
Instructor	Session 1	Session 2
Anxious	5.29 (0.24)	5.68 (0.25)
Confident	5.43 (0.24)	5.38 (0.30)

Problems correctly solved (H_3a_)		
Instructor	Session 1	Session 2
Anxious	9.89 (0.83)	11.65 (0.83)
Confident	8.73 (0.82)	10.28 (0.90)

Problems skipped (H_3b_)		
Instructor	Session 1	Session 2
Anxious	9.72 (0.81)	7.52 (0.72)
Confident	11.72 (0.90)	8.55 (0.94)

Table shows complete cell means in native units for the primary contrast effects of interest described in the text above and visualized in Figure 3. Values in parentheses are standard-errors of the mean.

#### Changes in learner state anxiety (H_2_)

Here, we tested whether changes in learner state anxiety depended on instructor paralinguistic cues (Figure 1, orange box). Results are shown in Figure 3a. We failed to find evidence that it does: *t*(82) = 1.37, *p* = 0.174, *d* = 0.30. While there was a marginal increase in state anxiety for learners in the anxious instructor condition (*p* = 0.07, *d* = 0.27), there was very little change in state anxiety for learners in the confident instructor condition (*p* = 0.840, *d* = −0.03). That said, the lack of a difference between the groups in changes scores (i.e., the lack of an interaction), and at best only a marginal change in the anxious instructor condition urges strong caution when interpreting these results. We thus view this result as, on the whole, failing to provide support for the Paralinguistic Model (specifically the orange box in Figure 1).

#### Math learning (H_3a_)

Here, we tested whether math learning (changes in the number of correctly solved items) depended on instructor paralinguistic cues (light green box in Figure 1). We failed to find evidence that it does: *t*(82) = 0.22, *p* = 0.829, *d* = 0.05. Encouragingly, learners in both the anxious instructor (*p* = 0.013, *d* = 0.38) and the confident instructor (*p* = 0.032, *d* = 0.35) conditions showed a significant improvement. On average, participants correctly solved roughly 7% more items after instruction. This is remarkable given that items were both open-ended (i.e., not multiple choice) and relatively challenging, and that all participants were relatively high in math anxiety. However, as this improvement did not differ as a function of paralinguistic condition, here again we failed to find evidence in support of the Paralinguistic Model (specifically the light green box in Figure 1).

#### Math avoidance (H_3b_)

We tested whether changes in computational math avoidance (changes in the number of skipped items) depended on instructor paralinguistic cues (dark green box in Figure 1). We failed to find evidence that it does: *t*(82) = 0.77, *p* = 0.452, *d* = 0.16. Encouragingly, learners in both groups showed a reduction in the number of items skipped (11.2% reduction on average). Learners who viewed the confident instructor showed a slightly larger reduction (13.2%, *p* = 0.001, *d* = 0.56) than those who viewed the anxious instructor (9.1%, *p* = 0.021, *d* = 0.35). However, difference between instructor conditions was not statistically significant. Thus, while results were highly encouraging from the perspective of reducing computational avoidance among HMAs, we nevertheless failed to find evidence in support of the Paralinguistic Model (specifically the dark green box in Figure 1).

### *Post hoc* analysis: time pressure

In this section, we examined whether anxious vs. confident paralinguistic cues’ impact on math learning and/or math avoidance depended on whether math problems were performed with or without time pressure. We entered the outcome variables from H3a and H3b (number of correctly solved items and number of skipped problems, respectively) into a 2(Session) × 2(Instructor Condition) × 2(Time Pressure) ANOVA. In each case, the critical effect was the three-way interaction, as this tests whether changes between sessions depended on both the instructor and time pressure. Cell means in native units are shown in Table 2.

**Table 2:** Mean math performance/avoidance by paralinguistic condition and time pressure condition.

Instructor	Problems correctly solved			
	Time pressure		Untimed	
	Session 1	Session 2	Session 1	Session 2
Anxious	5.37 (0.42)	5.87 (0.43)	4.52 (0.45)	5.78 (0.45)
Confident	4.50 (0.43)	5.23 (0.47)	4.23 (0.44)	5.05 (0.46)

Instructor	Problems skipped			
	Time pressure		Untimed	
	Session 1	Session 2	Session 1	Session 2
Anxious	4.70 (0.40)	3.72 (0.36)	5.02 (0.46)	3.80 (0.39)
Confident	5.75 (0.49)	4.48 (0.49)	5.98 (0.50)	4.08 (0.51)

Table shows cell means in native units for the *post hoc* analyses examining problems solved under time pressure vs. untimed. Values in parentheses are standard-errors of the mean.

#### Math learning

The critical three-way interaction was not significant: *F*(1,84) = 1.69, *p* = 0.197, *d* = 0.28. Participants on average completed more problems correctly under time pressure (*p* = 0.015, *d* = 0.32), and they showed marginally greater learning (improvement across sessions) when not under time pressure (*p* = 0.094, *d* = 0.19). Crucially for present purposes however, neither time–pressure effect differed based on instructor condition (*p*s > 0.19); that is, the influence of time pressure was not modulated by instructor paralinguistic cues.

#### Math avoidance

The critical three-way interaction was not significant: *F*(1,84) = 0.34, *p* = 0.565, *d* = 0.06. No other effect was significant, indicating time pressure had no effect on the number of items skipped, and, crucially, this was not modulated by instructor paralinguistic cues.

## Discussion

Given the individual and societal threats posed by instructor MA’s negative association with learner math performance, numerous studies have attempted to characterize the pathway connecting these nodes. While current explanations attribute performance outcomes to MA’s influence over either instructors themselves or the content provided to learners, the present study proposed a communication-based Paralinguistic Model for MA transference, connecting instructor math anxiety and learner math performance via paralinguistic cue usage, affective appraisals, and state anxiety changes. Here we tested several composite relationships of the proposed model via administration of an anxious vs. confident paralinguistic audio intervention. HMA adult participants completed assessments of instructor anxiety, their own state anxiety, as well as their own performance and tendency to avoid challenging math problems. Although results indicated our key manipulation was valid – the paralinguistically anxious instructor was rated as more anxious by learners – we found no evidence that this impacted learners’ state anxiety, math learning or math avoidance. Learners exposed to both paralinguistically anxious and confident instructors showed an improvement in performance and a reduction in avoidance; these improvements were greatest for the most difficult problems, and equivalent under timed and untimed conditions. In sum, this short, online math intervention succeeded in inducing math learning across multiple challenging math skills and contexts in highly math-anxious adults. Given poor adult numeracy in the United States especially among high-MA adults (OECD, 2024; Cates and Rhymer, 2003; Hart and Ganley, 2019), these results are broadly encouraging. They are perhaps disappointing for strong proponents of the Paralinguistic Model as outlined here. Conversely, our results may also indicate that online instruction may mitigate the negative impact of teacher-anxiety on math-anxious learners, though further work is needed to validate this latter assertion. Limitations and broader implications are discussed in greater detail below.

### Anxious math instructors were perceived as anxious, but this did not impact math learning

When introducing a new paradigm into the literature, it is important to first verify that the novel manipulation is operating as intended. To that end, Figure 2 shows that the current intervention was successful. The paralinguistically ‘anxious’ instructor was indeed rated as significantly more anxious by learners than the paralinguistically ‘confident’ instructor. Similarly, the paralinguistically ‘confident’ instructor was rated as significantly more confident than the paralinguistically ‘anxious’ instructor. From this perspective, the experimental stimuli operated as intended. Implicit emotional cues, and by extension emotional recognition more generally, emerge from interacting auditory and visual (e.g., facial expression) modalities (Ghaleb et al., 2023). Consequently, when one of these modalities is missing – as was the case in the present, audio-only intervention – emotional recognition accuracy has been shown to decline (Cao et al., 2014). The results in Figure 2 are therefore important in that they show the current paradigm was able to overcome this limitation.

The critical question, then, is whether the perceived anxiety of the instructor negatively impacted highly math-anxious learners. Prior work has indicated that speaker anxiety can impact learners in general (Sinclair and Ryan, 1987; Amiri, 2024). However, Figures 3a–c indicates that, at least in the current context, it did not. We observed no evidence that instructor anxiety reduced learning. This was true whether we examined changes across sessions (learning) or simply performance immediately following exposure to the instructor (S2 performance). Prior work has indicated that math anxiety may be especially deleterious for more challenging math problems (Ashcraft and Krause, 2007; Lyons and Beilock, 2012a,b) and when doing math under time–pressure (Ashcraft and Moore, 2009; Boaler, 2014; Chan and Tang, 2020; National Council of Teachers of Mathematics, 2023). Nevertheless, *post hoc* analysis (Table 2) showed that instructor paralinguistic cues had no specific impact on learning or avoidance under time pressure. In the next section, we examine why not.

### Anxious math instructors did not make highly math anxious learners more anxious

The paralinguistic model in Figure 1 hinges crucially on the idea that the perceived anxiety of the speaker (the math instructor in this case) will be transferred to – have a direct impact on – the anxiety state of the listener (the learner in this case). There is some work to support this idea (Sinclair and Ryan, 1987; Amiri, 2024). However, in the present study we found no such impact (Figure 3a). According to the Paralinguistic Model, failure to elicit higher anxiety in learners effectively ‘short-circuits’ the pathway, obviating progress through the remaining nodes of the model. In plainer terms, the anxious instructor did not negatively impact math learning because they did not elicit heightened anxiety in learners. That is not to say the highly math-anxious learners felt no anxiety when solving the math problems (prior research indicates they likely did; Daker et al., 2023). Rather, *their anxiety was not exacerbated by the anxious instructor* (relative to the confident instructor). From a strictly theoretical perspective, this result is perhaps disappointing for strong proponents of the paralinguistic model – or at least for those who would make strong claims about the ubiquity of its applicability.

From a practical perspective however, these results are perhaps somewhat encouraging. First, it suggests that if one can circumvent inducing a heightened state of anxiety in highly math-anxious learners, the subsequent chain of processes leading to poor math performance and learning outcomes typically seen in this group can be mitigated. While prior intervention work has indicated this is the case for math performance (Sammallahti et al., 2023), evidence supporting the idea in the context of *math learning* is limited. The current work may thus be taken as preliminary, indirect evidence that if one avoids exacerbating state anxiety in highly math anxious learners, their math learning may, to an extent, be preserved (The next section provides a more detailed discussion of the current paradigm as a means of inducing adult math learning in a math anxious population).

Second, from a math instructor’s perspective, allowing one’s anxiety to ‘show through’ may not be entirely disastrous for one’s students – even those ostensibly most sensitive to math anxiety. Elementary and early childhood educators tend to be especially prone to high math anxiety (Hembree, 1990), and more recent work has suggested that this anxiety may be transmitted to students with insalubrious consequences (Beilock et al., 2010; Hadley and Dorward, 2011; Ramirez et al., 2018; Richland et al., 2020; Schaeffer et al., 2021; Szczygieł, 2020). Perhaps ironically, awareness by teachers of this research may compound anxiety about *teaching* math (Stoehr, 2017). The current work therefore provides a degree of relief – showing that the link between a teacher’s anxiety and poor student outcomes is not ineluctable.

There are several potential explanations for why the current results differ somewhat from previous work on the transmission of math instructor’s anxiety. One explanation is that the current sample focused only on those high in math anxiety and low in math ability. However, prior work indicates students with high math anxiety are sensitive to teacher anxiety levels (Beilock et al., 2010; Hadley and Dorward, 2011; Ramirez et al., 2018; Richland et al., 2020; Schaeffer et al., 2021; Szczygieł, 2020; Kyttälä and Björn, 2010; Ma and Xu, 2004; Sorvo et al., 2019; Szczygieł et al., 2024; Zhang et al., 2023). A second possibility is that participants here were all adults, whereas prior research has focused mostly on elementary school students. Adults are generally more capable at emotion regulation than children (Cracco et al., 2017; Doerwald et al., 2016; Le Vigouroux et al., 2017; Shiota and Levenson, 2009), and so adult learners may be more robust to teacher anxiety. A counterpoint is that instructor anxiety can influence anxiety levels even in adults (Sinclair and Ryan, 1987), and math anxious adults show elevated state anxiety both when doing (Daker et al., 2023) and even considering the possibility of doing math (Lyons and Beilock, 2012a,b). Moreover, even if the adult cohort managed to successfully regulate state anxiety changes, the additional cognitive load incurred by this process would’ve likely counteracted any positive learning outcomes (Scheibe and Blanchard-Fields, 2009), further reducing the likelihood of this explanation. A third possibility is that the current learning environment was online, and instruction was presented via a recorded audio, not a live lecture or discussion. This asynchronous, audio-only presentation likely reduced the instructor’s capacity for anxious affective coercion (Suen and Hung, 2025) while also mitigating baseline social anxiety via an absence of in-classroom factors like social embarrassment. Relatedly, a person may be able to complete an online study in a more familiar and comfortable environment, which can further reduce state anxiety (Altıntaş and Meriç, 2024; Çiftçi and Öztunç, 2015; Dehghan et al., 2014; Şahan et al., 2024). Our tentative view is that this third explanation is most plausible, though it is crucial to note that the current work cannot show this conclusively because we did not have a live or in-person condition for comparison. Nevertheless, our view is that the current work suggests a promising and potentially provocative avenue for future intervention work: Digital or online learning environments may prove protective for both instructors and learners suffering from high levels of math anxiety, much in the same way that previous work has suggested they help with socially anxious learners (Abramson, 2021; Yen et al., 2012).

### Highly math anxious adults learned math

Average adult numeracy in the United States is poor (National Center for Education Statistics, 2024), and at the same time contemporary economic changes have largely favored those who have – or can rapidly develop – strong numeracy skills (Durrani and Tariq, 2012; Vignoles and Cherry, 2020). High math anxiety is associated with both short and long-term avoidance of math (Daker et al., 2021; Lau et al., 2024). A lifetime of avoiding math can lead to declining math skills, which in turn is predictive of lower income, poorer health, and accelerated cognitive decline (Crawford and Cribb, 2013; Ritchie and Bates, 2013; Reyna et al., 2009; Garcia-Retamero et al., 2015; Reyna and Brainerd, 2007; Heilmann, 2020; Hanushek et al., 2025). Conversely, consistent engagement and practice with math can preserve and enhance basic math skills (Reder et al., 2020; Fioriti, 2023). There is a critical need for low-cost delivery of effective instruction in basic numeracy (OECD, 2024; Marcus, 2023).

The current paradigm demonstrates that short (<30 min), targeted math instruction delivered in a pre-recorded online format can lead to math learning across a variety of basic numeracy skills. This learning was also observed under time–pressure, indicating this learning may generalize to high-pressure situations. What is remarkable is that the largest gain was in fact a reduction in the number of skipped items. After instruction, participants attempted an average of 11% more items. Put another way, participants were less likely to avoid – or perhaps more likely to engage with – roughly a quarter more math problems after instruction. This effect was strongest for the types of problems (systems of equations and algebraic reduction) participants were likely least familiar with. Crucially, this reduction increased willingness to engage with unfamiliar math was observed in individuals for whom increasing math engagement and learning is especially critical – those with high math anxiety and poor math skills. Finally, this learning was invariant with respect to instructor anxiety. As noted in the previous section, we suspect this last point may be due to the pre-recorded, online delivery mode. While further work is needed to verify this claim, if correct, it would point toward a low-cost means of improving adult numeracy among those most at risk for poor numeracy-related outcomes.

### Limitations and mitigating arguments

One potential limitation of the current work is that the primary instructional and learning portions of the study limited participation to individuals above average in terms of math anxiety (HMAs) and below average in terms of math performance. This was because these low-performing HMAs presented the greatest need for efficacious math instruction, and thus stood to gain the most from the present work’s findings. Further, per the Introduction, we expected that HMAs would be most sensitive to paralinguistic instructional cues, at least relative to their LMA counterparts (Beilock et al., 2010; Hadley and Dorward, 2011; Ramirez et al., 2018; Richland et al., 2020; Schaeffer et al., 2021; Szczygieł, 2020; Kyttälä and Björn, 2010; Ma and Xu, 2004; Sorvo et al., 2019; Szczygieł et al., 2024; Zhang et al., 2023). While these rigid selection criteria allowed us to examine the tested interventions across a particularly impactful subpopulation, we also recognize that they limit the generalizability of our findings. Moreover, given the low baseline performance across our final sample, it remains possible that observed learning effects were partially the product of said performance deficits. That said, future work may examine whether paralinguistic cues impact learning in low math anxious individuals and/or high-performing differently.

Another potential limitation is that the current study did not include a ‘business as usual’ condition. Thus, we cannot say for certain that the learning we observed was strictly caused by the instructional video (recall that we observed significant learning for both paralinguistic conditions). While this is an important caveat to bear in mind, we find specific alternative explanations to be less plausible. First, specific items were not repeated between sessions, so individuals could not have memorized the answers to specific items. It is possible that practice effects might nevertheless account for accuracy improvements on the items participants attempted. However, it is harder to see how practice effects explain the significant reduction in the number of items participants skipped. If an item was skipped, then by definition it was not practiced. In our view, the more parsimonious explanation is that the instructional videos provided concepts and strategies that made participants more likely to attempt previously skipped items, and to improve their accuracy on those they previously attempted as well.

It is also important to note that the current participants were adults. Improving adult numeracy is, we have argued, an urgent matter worthy of greater study. That said, as noted above, children may be more readily influenced by teacher paralinguistic cues. However, this is an empirical question, which would require direct investigation before drawing conclusions about the relevance of this work to children’s development.

A final limitation to note is that these data were collected in an online setting. This was done in part to ensure a more representative sample than is often available in a typical college campus research setting. A downside of this approach, as we have argued above, is that paralinguistic cues may operate differently in person. Because adult learning in particular increasingly occurs in online settings, we believe the current results remain highly relevant. However, further work is needed to assess whether these results extend to in person settings.

## Conclusion

The present work offers a set of optimistic takeaways surrounding adult-oriented math education. Of primary note is the discovery that, even among highly math anxious adults, teachers’ use of anxious paralinguistic cues in their instruction did not prevent these learners from either (A) improving their math skills or (B) becoming less avoidant toward math. Rather, learners were able to leverage learned concepts toward attempting and successfully completing more problems, regardless of the detected anxiety/confidence in their teacher’s voice. This finding was also found to hold up under high-pressure situations (i.e., time-pressured problem solving), and was uncovered within an online learning context, making it especially relevant to the increasingly digital world of adult education. These findings are doubly beneficial, not only alleviating the fears of math-anxious adult educators (i.e., that their anxiety will “seep through” into their instruction and harm their learners), but also pointing toward a potential low-cost medium for adult numeracy education in a day and age where it is crucially relevant to the development of society and its constituents.

## Data Availability

The raw data supporting the conclusions of this article will be made available by the authors, without undue reservation.
