# Supplementary material for: Testing whether paralinguistic cues alter math learning in highly math anxious adults
Source: Front Psychol. 2026 Jun 17;17:1752172. doi: 10.3389/fpsyg.2026.1752172 (PMC13318949; doi:10.3389/fpsyg.2026.1752172)
Supplement: Supplementary file 1 [file Supplementary_File_1.docx]

**APPENDICES**

**Appendix A: Paralinguistic Instructional Video – General Instructions**

The following instructions were shown in written form just prior to starting the instructional video. The same instructions were given for both conditions (Anxiety and Confidence).

You are about to listen to an audio recording containing instructions for a number of math problem types.

Please listen to the entirety of the recording only once.

Please ensure that you are alone and in a quiet space while listening to the recording.

Please ensure that external distractions are minimized while listening to the recording.

Please do not alter the playback speed of the recording.

You may take notes during the recording if you wish, but please do not pause the recording at any point once you begin listening.

Your time spent on the following page will be recorded, in order to verify that you began listening to the recording promptly, did not alter its playback speed, only listened to the recording once, and did not pause the recording over the course of its playtime.

Once you have finished listening to the recording, a button will appear on the bottom right corner of the screen. Click it to continue onto the following task.

Click the button below to continue onto the recording.

**Appendix B: Paralinguistic Instructional Video – Full Script**

The following is the full script used for both the Anxious and Confident versions of the instructional math video(s).

*[Introduction]*

“Hello, and thank you again for your participation in the study. You are about to complete a brief assessment of your mathematical abilities. You will be exposed to a range of problem types, gauging multiple skills and spanning across multiple degrees of difficulty.”

*[Whole-Number Arithmetic]*

“The first phase of the task will gauge arithmetic operations, including addition, subtraction, and multiplication. You may be asked to operate on whole numbers or fractions. As a reminder, addition problems require that you sum together two or more values, appending each new value to the running total until you reach a final sum. For example, the expression [3 + 5 + 2] asks you to take the value of 5 and append it onto the value of 3, yielding an added sum of 8, then to take the value of 2 and append it onto the added sum of 8, yielding a final added sum of 10.

“Subtraction, on the other hand, requires that you remove the value of a latter total from that of its formerly-presented counterpart. For example, if you are presented with the expression [15 - 7], you would need to take the initial value of 15 and take away the secondary value of 7 from it, yielding a final subtracted sum of 8.

“Multiplication problems require you to sum together a set number of instances of a presented value in order to yield a product. Think of it like repeated addition, where you add the first value to itself as many times as the second value dictates. For example, the expression [8 * 7] prompts one to sum together the value of 8, 7 times (or vice versa), yielding a final product of 56.

“When working on problems that combine these three arithmetic operations, it is important to remember the order in which these operations are supposed to be completed. In this case, you should always do multiplication-based parts of a given problem first, then complete any addition or subtraction-based parts in whatever order they are presented, from left to right. For example, if you are given the expression 4 + 2 * 3 - 1, you would start by doing the multiplication problem in the middle of the expression first. In this case, you would multiply 2 by 3 to find a product of 6. Now, your expression has become 4 + 6 - 1. Since the addition component comes first, you should then add 4 and 6 together to give yourself an added sum of 10. Now, your expression is 10 - 1, and using subtraction to complete the final step leaves you with a final total of 9.”

*[Fraction Arithmetic]*

“If, during these arithmetic operation problems, you are asked to work with fractions, many of the previously discussed rules still apply. There are, however, a few differences to keep in mind as you work through these problems. First, for fraction problems, you will only be presented with addition and subtraction problems to work through. Secondly, when you are adding or subtracting fractions from one another, you will sometimes run into cases where you are asked to add/subtract fractions that have different denominator values. Denominator values are the numbers that occupy the bottom line of a fraction, and if these numbers do not match when you are attempting to add/subtract fractions, you cannot complete these operations. When you run into one of these types of problems, you will need to manipulate the fractions in such a way that the denominator values are matching. Generally, this is done by finding what is called a least common factor for the denominator values that you are working with.

“A least common factor is the lowest number that is a shared multiple of all of the denominators that you are working with in a given problem. You can find this number by writing out of the multiples of your various denominator values, going down the line until you find a number that has appeared in every sequence. For example, if I am working with the denominator values 2, 3, and 4, I will start by writing out a few multiples of 2: 2, 4, 6, 8, 10, 12. Then a few multiples of 3: 3, 6, 9, 12, 15, 18. And finally, a few multiples of 4: 4, 8, 12, 16, 20, 24. Already, I can see that the number 12 has appeared in all three of my sequences, making 12 my least common factor (note: if you don’t see a common factor after this first round, just keep going until you do; on the contrary, if you see multiple, your least common factor should, as its name suggests, be the one with the lowest value). Once you have your least common factor, the next step is to convert your existing fractions into fractions with the least common factor as their denominator.

“The first step in doing this is to find out how many times your current denominator fits into the least common factor. For example, the denominator value 3 from my previous example fits into the least common factor of 12 4 times. From there, multiply the top and the bottom of the fraction by this value, with the resulting fraction being equivalent to your original fraction, just in its least common factored form. For example, if my fraction is ⅔, and I determine that my denominator of 3 fits into my least common factor 4 times, I would then multiply both 2 and 3 by 4 to get a top value of 8 and a bottom value of 12. The resulting fraction, 8/12, is equivalent to my original 2/3, just in least common factored form. Once you do this to all of the fractions in your problem, you can start adding and subtracting, using the normal rules.

“One trick for completing fractional arithmetic problems is to add and subtract the fraction components and the whole number components separately, then add them together at the end. For example, if I have 5 ½ - 2 ⅔, the process from start to finish would look something like this. Take the denominator values 2 and 3 and find their least common factor. A short list of 2s multiples is 2, 4, 6, 8, 10, and 12, while 3s are 3, 6, 9, 12, 15, and 18. The smallest number present in both of my lists is 6 in this case, making it my least common factor. Now, to convert the fractions ½ and ⅔ into fractions with 6 as their denominator. 2 fits into 6 3 times, so I'll multiply the top and the bottom of ½ by 3 to get the fraction 3/6. 3 fits into 6 2 times, so I'll multiply the top and bottom of ⅔ by 2 to get the fraction 4/6. I can then subtract 4/6 from 3/6, to get a final subtracted fraction total of negative ⅙. Now for my whole numbers, I simply take 2 and subtract it from 5 to get a final subtracted whole number total of 3. I then have to add negative ⅙ to 3, which is the same thing as subtracting ⅙ from 3. When it comes to subtracting fractions from whole numbers, it helps to think about it like a pie that you’re taking away pieces from. For example, if you have three whole pies, and take away a 1/6th slice from one of them, you’d be left with 2 whole pies and 5/6ths of the third pie leftover, leaving you with 2 5/6th pies. Similarly, if you subtract ⅙ from 3, you are left with the final answer of 2 ⅚.

“One last thing to keep in mind when adding and subtracting fractions is what to do when you have a very large fraction. That is, a fraction where the number on top (the numerator) is bigger than the number on the bottom (the denominator). In these cases, subtract the denominator from the numerator as many times as you need to until the denominator is the bigger number. Then, count how many times you had to subtract the denominator from the numerator and add/subtract that count to/from your whole number total, leaving the leftover fraction as your fraction total. For example, if I am left with the fraction 21/4, I’ll subtract four from 21 as many times as I need to until 4 is bigger than the number on top. In this case, I’d have to subtract four from 21 5 times, leaving me with a fraction total of ¼. From there, I would take my 5 count and either add it to or subtract it from my whole number total, depending on the type of operation I’m working with.”

*[Systems of Equations]*

“The third phase of the task will gauge your ability to solve a system of equations with two unknowns. To make this explanation simpler, let's say our two unknown variables are x and y, and our system of equations includes the equations x+ 2y = 5 and 3x - y = 1. In order to do this, you must first isolate one of the variables in one of the equations, such that it is set equal to a whole number value and multiples of the second variable. In our example, we can take the equation x + 2y = 5 and isolate x on one side of the equation by subtracting 2y from either side. This leaves us with the equation x = 5 - 2y. Following this, you must find every instance of your isolated variable in the second equation and replace it with the expression from the first equation. For our example, we would take our second equation of 3x - y = 1, and replace every x with 5-2y, the expression we set x equal to in the first equation. With this change, our second equation becomes 3(5-2y) - y = 1, which expands out to 15 - 6y - y = 1, or 15 - 7y = 1.

“From here, you should solve this newly simplified second equation, which will end up with you solving one of the two unknown values in your system of equations. For our example, we would solve 15-7y = 1, which can be simplified out to -7y = -14, which can be divided by -7 on both sides to yield y = 2, solving one of our unknowns. From here, simply plug your newly solved unknown value into either original equation to solve for the second unknown value and complete the system. For our example, we can plug in 2 for y in the original equation x + 2y = 5 to yield the equation x + 2(2) = 5. Distributed out this becomes x + 4 = 5, and if we subtract four from both sides, we get x = 1, solving our second unknown and completing our system.”

*[Reduction of Expressions]*

“The final phase of the task will gauge your ability to reduce expressions composed of unknown variable values. Note that for these problems, your task is not to solve for a variable value, but rather to reduce the expression you are given to its simplest form. While there is no universally-applicable way to do this (at least not one that can be covered in the span of this recording), there are a few strategies that may assist you in arriving at a final, simplified solution.

“The first is to look for common factors in fractional expressions that can be removed. For example, if one of the expressions contains the fraction 3X/X, the common factor of X can be removed from both the numerator and the denominator, yielding a simplified expression of 3/1, or 3. Similarly, if an expression has 3x+3 on top and 6 on the bottom, the common factor of 3 can be removed from the numerator and the denominator, yielding a simplified expression with x+1 on the top and 2 on the bottom.

“The second tip would be to look for certain forms of quadratic expressions that have common and well-known factors that they can be simplified down to. There are three of these quadratic forms that you should keep in mind while you do your problems. The first is [a-squared + 2ab + b-squared], which can be expanded into (a+b) * (a+b). For example, the quadratic equation x-squared + 6x + 9, which has x and 3 in place of a and b, can be simplified down to (x+3) *(x+3). The second quadratic form is [a-squared - 2ab + b-squared], which can be expanded into (a-b) * (a-b). For example, the quadratic equation x-squared - 4x + 4, which has x and 2 in place of a and b, can be simplified down to (x-2) * (x-2). The third and final common quadratic form, [a-squared - b-squared], can be expanded into (a+b) * (a-b). For example, x-squared - 16, which has x and 4 in place of a and b, can be simplified down to (x+4) * (x-4). An additional note for working with these kinds of quadratics is to remove any common numerical factors before you try to simplify them down. For example, if you have the quadratic expression 3 x-squared + 24x + 48, make sure to set aside the common factor of three first, which will leave you with 3 times the expression (x-squared + 8x + 16), which can then be simplified down to 3 times the expression (x+4) * (x+4).

“A third useful tool for reaching your final, reduced expressions can be found in the concepts of fraction multiplication and division. When doing fraction multiplication, it is important that you remember to multiply the numerators by one another to get the final fraction’s numerator, and multiply the denominators by one another to get the final fraction’s denominator. When dividing fractions, the process is the same, but prior to multiplying, you should flip one of the fractions, such that its numerator is now its denominator and vice versa. For example, if you are dividing x over 2 by x over 3, it is the same as multiplying x over 2 by 3 over x. It is often best to do this multiplication/division step last, after you have already expanded out your quadratic forms and dealt with any common factors. As a final note, remember that if you are multiplying or dividing fractional expressions by one another, any common factors that apply to one also apply to the other. This means that if you have, for example, an (x+2) factor in the numerator of one fraction and the same (x+2) factor in the denominator of the other fraction, you can cancel those factors out, removing them both from the overall expression. Once you have completed these steps, you will ideally have simplified your expression completely. You will know an expression is simplified completely when the numerator and the denominator share no remaining common factors.”

*[Conclusion]*

“Now that we have covered the main phases of the math task you are about to complete, you will be given an opportunity to demonstrate your capacity for these skills by completing the task. Continue on to the next section by clicking the button below.”

**Appendix C: Math Problem Examples**

Basic Arithmetic Problems

| - 7 x 8 - 19 = ? |
| --- |
| - 9 x 7 - 17 = ? |

Fractional Arithmetic Problems

| - 9 1/3 + 2 1/4 + 3 1/8 = ? |
| --- |
| - 8 2/3 - 2 1/8 = ? |

Systems of Equations Problems

| - x+ 4y = 36 ; 2x - 3y = -16 \| x = ?, y = ? |
| --- |
| - 2x - 3y = 11; x + 4y = 11 \| x = ?, y = ? |

Expressional Reduction Problems

$$\frac{2P+4}{4P}\cdot\frac{2P^{2}-4P}{P^{2}-4}= ?$$

$$\frac{3P^{2}+6P}{6P^{2}}\cdot\frac{4P+8}{{2P}^{2}+8P+8}= ?$$

**Appendix D: General Math Task Instructions**

The following instructions were shown in written form just prior to starting the math task. These same instructions were shown in both Sessions 1 and 2.

For the following task, you will be asked to answer questions gauging a number of mathematical skills. Please attempt to answer all questions. That being said, if you cannot determine the answer to a question, please enter "NA" in that question's response box.

For questions where more than one answer is prompted, please respond with a list of your answers (e.g., x = ?, y = ?).

For questions with a fractional answer, enter your answer using the '/' symbol and spaces as needed (e.g., 7 3/4, 8/9, x/2, 1/x, 3x/2, 9 and 1/4, etc. are all acceptable answer forms)

Please refrain from using a calculator or any other assistive problem solving tool for this task. That being said, you are more than free to use a pencil and paper to work through the problems as you wish.

Note: throughout these problems, multiplication is represented using the dot operator ( ⋅ ), such that (9 ⋅ 3) is equivalent to (9 x 3)

Press the arrow below to continue.
